# Measuring the Possibility of Middle Ear Discharge for COVID-19 Test Material

**DOI:** 10.1155/2022/7207846

**Published:** 2022-04-20

**Authors:** Hamsu Kadriyan, Lalu Hizrian Rizkika Abtartu, Eustachius Hagni Wardoyo, Fahrin Ramadan Andiwijaya

**Affiliations:** ^1^Department of Otolaryngology Head and Neck Surgery, Faculty of Medicine, University of Mataram, Mataram, Indonesia; ^2^Department of Microbiology, Faculty of Medicine, Mataram University, Mataram, Indonesia; ^3^Department of Public Health, Faculty of Medicine, University of Mataram, Mataram, Indonesia

## Abstract

The COVID-19 pandemic is still ongoing, and new variants continue to emerge. Various examination methods and sampling specimens are continuously being developed and published. The standard for sampling is in the nasopharynx. However, in children, this is often uncomfortable and at risk of eliciting complications. Therefore, it is necessary to look for other alternative sampling sites such as fluid from the middle ear. Scientific evidence shows that the middle ear can be a place for the attachment and growth of the SARS-CoV-2 virus. Currently, to the best of the author's knowledge, there have been no publications on middle ear discharge as a sample for the determination of the diagnosis of COVID-19. Based on this, the authors would like to explore the possibility of middle ear discharge for COVID-19 test material. A narrative review on the use of middle ear discharge as a potential diagnostic specimen for COVID-19 was conducted. The searches were conducted in the PubMed and ProQuest databases.

## 1. Introduction

The COVID-19 pandemic has been ongoing for the last 3 years. Moreover, the COVID-19 pandemic is marked by the emergence of several new variants of concern such as alpha, beta, gamma, delta variants [1], and the latest is the omicron variant, whose transmission is faster than the previous variant [2]. Various diagnostic methods have been developed to speed up and facilitate the determination of the diagnosis of the disease. The standard method to date is the use of reverse transcription-polymerase chain reaction (RT-PCR) taken from nasopharyngeal swab material [3].

For children, a nasopharyngeal swab can be an uncomfortable procedure. Furthermore, this action also has the potential to cause complications such as epistaxis, and even intracranial complications can occur [4, 5]. Therefore, it is necessary to continue to look for a more simple, safe, and convenient sampling location to improve the COVID-19 diagnosis.

The middle ear is anatomically connected with the nose and nasopharynx via the eustachian tube, so that if there is a malfunction of the eustachian tube this can trigger an infection in the middle ear or otitis media [6]. Cases of otitis media are still quite common and cause a fairly high economic burden and affect the quality of life of patients and their parents [7]. Otitis media is more common in children than in adults [6]. In children aged 3 years, more than 50% have experienced at least 1-time otitis media, and the peak incidence is at the age of 6–12 years [8]. One of the signs of a middle ear infection is the discharge produced. This discharge can then be taken as material for various examinations [6]. In this paper, we will discuss the potential use of middle ear secretions or discharge in otitis media patients, especially in children, to be used as material for the diagnosis of COVID-19.

## 2. Methods

In this study, the authors used a narrative review method. The search was conducted in the PubMed and ProQuest databases, using three main concepts: (1) COVID-19 diagnostics, (2) middle-ear discharge, and (3) otitis media. All articles that have been published in the peer-reviewed journals on both databases and related to those concepts were included in the analysis. Finally, a conclusion was made based on the result of the analysis [9].

## 3. The Relationship between the Nasopharynx and the Middle Ear

Anatomically, the middle ear has a very close relationship with the nasopharynx. These two structures are connected directly by the eustachian tube [10]. Histologically, the epithelium in the middle ear is similar to that in the nasopharynx and eustachian tube, i.e., both have ciliated stratified epithelium. Although the cilia in the middle ear and eustachian tubes move towards the nasopharynx, in certain conditions this can be disturbed. Eustachian tube dysfunction is defined as the manifestation of unregulated middle ear pressure [11].

Upper respiratory tract infections often result in middle ear infections. This is caused by impaired ciliary function in the eustachian tube and impaired tubal patency [12]. In addition, positive pressure, such as performing the Valsalva maneuver, may also provoke impaired ciliary function where the pressure actively sends fluid from the nasopharynx to the middle ear. This allows the migration of bacteria or viruses from the nasopharynx to the middle ear. Finally, this can lead to an infection in the middle ear. According to Jeong et al. (2021), the spread of COVID-19 through the eustachian tube is one way of spreading the infection to the inner ear [13]. Fidan first reported a case of otitis media in 2020 in a COVID-19 patient [14]. Raad et al. (2021) reported 8 cases of otitis media in COVID-19 patients who had no history of middle ear infection. One patient was found to have a tympanic membrane perforation [15].

A three-year multicenter study in Spain found 521 cases of spontaneous tympanic membrane rupture due to acute otitis media in 478 pediatric patients aged 2–8 years [16]. Tympanic membrane perforation is generally followed by discharge from the middle ear to the outer ears. During the lockdown period in Italy, 343 patients with otitis media were found out of 5438 patients, 62 of whom experienced spontaneous tympanic membrane perforation [17].

## 4. Otitis Media and Middle Ear Secretion as a Medium for the Growth of Microorganisms

Middle ear infections are divided into several classifications. Based on the course or duration of the disease, middle ear infections are divided into acute and chronic otitis media. Acute otitis media, if the occurrence is less than 3 weeks [18], while chronic otitis media, if it lasts more than 2–6 weeks [19]. If at least three episodes of acute otitis media for 6 months or 4 times in 1 year have occurred, then recurrent acute otitis media may also occur [20].

Perforation can occur in acute middle ear infections and can last for months or even years. Based on the location of the perforation, middle ear infections can be divided into central, subtotal, total, and marginal perforations. The location and size of the perforation can indicate the possibility of a safe or dangerous type of infection [21, 22].

Otitis media with perforation is usually followed by a discharge from the middle ear. Persistent perforation will facilitate the occurrence of reinfection that triggers the discharge of fluid again. Reinfection is often associated with inflammation or upper respiratory tract infection. It can also be caused by the contamination of fluids from outside [22]. Thus, both acute otitis media with perforation and chronic otitis media have the potential to spread the COVID-19 virus from the nose and nasopharynx to the middle ear or even directly inoculate the middle ear through the tympanic membrane perforation.

Middle ear discharge, similar to tears, saliva, and cerumen, can be a place for the COVID-19 virus to be found [23, 24]. However, the routine use of middle ear fluid in the diagnosis of COVID-19, especially in children, has not yet been established. In addition, the procedure for obtaining the middle ear discharge in otitis media is simple and widely used to detect many kinds of microorganisms [25, 26]. Complications may arise during this procedure including discomfort and bleeding [24].

## 5. Receptors in the Middle Ear Associated with COVID-19 Infection

Cellular entry and replication mechanisms of SARS-CoV-2 in human cells were explained by Senapati et al. (2021) as follows: SARS-CoV-2 enters the cell through aerosol transmission and binds to the ACE2 receptor, which mainly disperses in the alveolar cells of the human lungs and fuses with the membrane. This requires the two domains of S1 and S2 of spike (S) protein to be cleaved using TMPRSS2 (serine proteases) [27].

The FURIN gene has a direct regulatory function, including in cell differentiation, protein cleavage, and cell invasion. Unsupervised pathway assessment showed that TMPRSS2 is implicated in several biological pathways involving cell fusion, viral entry, and vascularization, in addition to being associated with severe acute respiratory syndrome pathogenesis, along with ACE2. ACE2 is an upstream regulator for several of the identified interactomes involving viral reproduction and the inflammatory response to viral infections [28]. A study in Spain found the TMPRSS2 gene was most likely associated with SARS-CoV-2 infection compared to the ACE2 receptor gene and FURIN gene [29].

In the head and neck region, it was concluded that ACE2 and TMPRSS2 were found to be distributed in humans, monkeys, and mice. This illustrates the possibility of COVID-19 infection in the head and neck area [30]. In a study with 38 adult participants, cerumen, tears, and saliva specimens were taken concurrently with oropharyngeal and nasopharyngeal swabs There was a decrease in the positivity rate in saliva 76.3%, tears 55.3%, and cerumen 39.5% compared to the nasopharyngeal-oropharyngeal swab [23].

In mice, ACE2 was found in the epithelium of the middle ear, eustachian tube, and inner ear. TMPRSS2 was found especially in the mucosal epithelium of the middle ear and eustachian tube as well as the organ of Corti. FURIN was expressed in the middle ear, eustachian tube, and cochlea cytoplasm [31]. In the human inner ear, based on the in vitro cellular model, the levels of ACE2, TMPRSS2, and FURIN were higher than in the control group [13]. A study in rats demonstrated that ACE2 and TMPRSS2 were dispersed along the nasal cavity, larynx, and lungs [32].

Frazier et al. (2020) found SARS-CoV-2 in the middle ear and mastoids. According to this report, the virus was expressed in 2 of 3 patients (75%) [33]. The COVID-19 examination of the middle ear of the corpse found 50% positive for SARS-CoV-2 with a CT value of 17–31. When compared with nasal swabs, the proportion of PCR for COVID-19 in the middle ear is 66%, even COVID-19 can be found in the ear even though there is no evidence of previous middle ear infection [34]. Indirectly, middle ear disorders due to COVID-19 are shown by hearing loss [35]. In vitro studies of SARS-CoV-2 inoculation in the middle ear mucosa were well demonstrated, as indicated by the expression of N1 and N2 proteins [36].

## 6. Pathogenesis of Viral Infection in Otitis Media

Various types of viruses that cause upper respiratory tract infections can induce acute otitis media, including respiratory syncytial virus (RSV), rhinovirus, adenovirus, coronavirus (including COVID-19), bocavirus, influenza virus, parainfluenza virus, enterovirus, and human metapneumovirus [15, 37, 38]. Viral infection can induce the changes of nasopharyngeal mucosa through modification of host immune function [39], promote cytokine activity and proinflammatory mediators [40] and increase the colonization and adherence of bacteria through the upregulation of host cell surface antigens that serve as bacterial receptor sites [41].

Viral infection promoted the changes in mucus character and disturbed the normal mucociliary clearance in the eustachian tube and nasopharynx. This will lead to eustachian tube dysfunction and promote negative middle ear pressure [42]. Negative middle ear pressure will facilitate the influx of bacteria and/or viruses into the middle ear [12]. Independent viral infections can cause acute otitis media [38]. However, most acute otitis media occurs after a symptomatic viral upper respiratory tract infection [43]. Where, about 27% of acute otitis media is related to upper respiratory viral infection, especially in those who are under 1 year old [37]. In the patient with serous otitis media, polyomavirus, one of the common upper respiratory viral etiology, could be detected in the middle ear fluid [44]. In the case of COVID-19, direct inoculation of SARS-CoV-2 in the middle ear was documented [36].

With the presence of receptors in the middle ear and evidence of COVID-19 inoculation in the middle ear, there is a possibility that COVID-19 can cause further infections in the middle ear. This situation may then result in the formation of fluid in the middle ear [22]. Infection in the middle ear can be an infection with or without perforation of the tympanic membrane [6, 19]. Middle ear infection with perforation results in fluid leaking from the middle ear into the outer ear [19, 22]. The fluid produced in the middle ear may contain the virus or part of the COVID-19 virus.

## 7. Advances in the Diagnosis of COVID-19

Han et al. (2021) divide the COVID-19 detection technology broadly into nucleic acid-based, serological-based detection methods, and CT imaging-assisted diagnosis [45]. However, another study by Etiene et al. suggested that detection of COVID-19 can be categorized into viral antigen and antibody [46]. Nucleic acid-based detection methods include real-time RT-PCR, digital polymerase chain reaction (dPCR), metagenomics next-generation sequencing (mNGS), reverse transcription‐loop‐mediated isothermal amplification (RT-LAMP), and clustered regularly interspaced short palindromic repeats (CRISPR). Each of these methods has advantages and disadvantages, but in terms of time, the fastest result is CRISPR followed by RT-LAMP and the longest is mNGS (Table 1) [45–47]. Syamsun et al. (2022) used a rapid molecular testing (RMT) cartridge to detect the COVID-19 nucleic acid in corpses (Table 1) [48].

Serological-based detection methods include colloidal gold immunochromatographic assay (GICA), chemiluminescence enzyme immunoassay (CLIA), enzyme-linked immunosorbent assay (ELISA), and lateral flow immunochromatographic assay (LFIA). Each of these technologies also has advantages and disadvantages, but in terms of the speed of obtaining results, GICA is the fastest, followed by LFIA, while ELISA is the longest (Table 1) [45]. The antigen-based test is also reliable for rapid COVID-19 diagnostics, with a sensitivity of around 85 to 95% [49].

**Figure 1:**
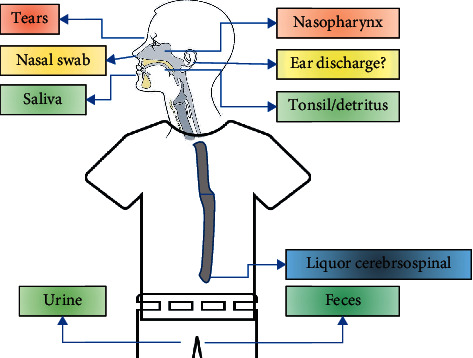
The sample collection site for the diagnosis of COVID-19.

Likewise, the location of sampling collection is increasingly varied, such as from saliva, rectal secretions, urine, blood, stool, and others (Figure 1) [50–54]. Even materials that are in the environment can also be a source of inspection such as water in a septic tank [55].

## 8. Timing and Potential Scoring System to Use Middle Ear Discharge for Diagnosing COVID-19

A nasopharyngeal swab to detect SARS-CoV-2 is recommended for patients with COVID-19 symptoms. Moreover, it is also indicated for children who are in close contact with probable cases or confirmed positive patients, and for those who require hospitalization or in case of traveling purposes. The swab is not recommended in children with symptoms that have a known infectious focus and who do not need hospitalization. [56].

**Figure 2:**
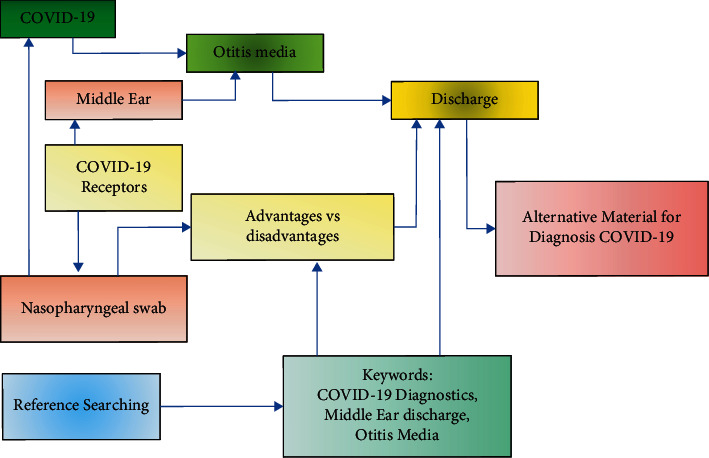
Schematic diagram on measuring potency of the middle ear discharge to be alternative material for COVID-19 diagnosis.

Based on the previous explanation, Table 2 shows the summary of the comparison between nasopharyngeal and middle ear discharge swabs for the diagnosis of COVID-19. In advance, the summary of how the middle ear discharge could be used as a specimen for establishing the COVID-19 diagnosis is shown in Figure 2.

The basic considerations regarding whether which type of body fluid such as middle ear discharge can be used as an alternative specimen for the diagnosis of COVID-19 are as follows: (1) the pathogenesis of COVID-19 is the cause or result of the production of more excreta body fluids; (2) middle ear secret can be a good virus transport media; (3) the causative pathogen was found in large numbers exceeding the minimum viral detection threshold; (4) the main specimens (nasopharyngeal swabs and nares swabs) were difficult to collect, especially in pediatric patients; and (5) there has been a perforation of the tympanic membrane.

Based on the explanation above, we propose the following scoring system to increase the possibility of middle ear fluid as an alternative diagnosis of COVID-19: (1) perforation of the tympanic membrane was found; (2) adequate middle ear fluid is found in the external ear; (3) there is a respiratory infection that causes otitis media. Each point is given a value of 1 if it finds the sign and 0 if it is not found, except for point 3. If it is not found, it is given a value of -1. If the sum result is 3 points, then the middle ear fluid is recommended to be used as a sample for COVID-19 testing.

## 9. Conclusion

Based on the review of this article, middle ear fluid has the potential to be used as an alternative sample for the diagnosis of COVID-19, but several things must be considered to get better results. Direct research aimed at this purpose is needed to determine with certainty the sensitivity and specificity of middle ear fluid in the diagnosis of COVID-19.

## Figures and Tables

**Table 1:** Profile of methods for diagnosis of COVID-19.

Methods	Advantage (s)	Disadvantage (s)	Classification according to speed for obtaining the result	
Nucleic acid-based	RT-LAMP	Easy and simple to operate and high sensitivity and specificity	More risk of false positive or negative results	1
	RMT	Easy to operate and cost-effective	Sensitivity lower than RT-PCR	2
	RT-PCR	The gold standard for diagnosis of COVID-19 and high sensitivity and specificity	Not only live virus could be detected but also part of the virus	3
	dPCR	Performs better than RT-PCR	High cost	4
	mNGS	The best methods for detection of the pathogen genome	Detected genome is limited and may contain nonspecific genome sequences	5
Serological-based	LFIA	Simple and low cost	Low sensitivity	2
	GICA	Simple and easy to do the test	High false positive	1
	CLIA	High sensitivity and specificity	High false positive	3
	ELISA	High sensitivity and specificity	Possibility of contamination	4
Antigen-based		Simple and quick	Less sensitive than RT-PCR	
CT imaging-assisted		Specific finding: ground-glass opacity and consolidation	Not used for determining diagnosis of COVID-19	

**Table 2:** Comparison of nasopharyngeal swab and ear discharge swab for COVID-19 diagnosis.

Methods	Nasopharyngeal swab	Ear discharge swab
Viral load	Highest	High in the middle ear
Receptor COVID-19	++	+
Convenient or ease of obtaining a sample	Less convenient (especially for children)	More convenient
Use in children	Recommended for those who are undergoing in-patient treatment	Currently, no data
Complication	Nasal bleeding and the risk of intracranial injury	Discomfort and bleeding

## Data Availability

The data supporting this NARATIVE REVIEW are from previously reported studies and datasets, which have been cited. The processed data are available in PUBMED and PROQUEST databases.
